# Particular Water Organization in Homogeneous Water Mixtures of Amphiphilic Polystyrene-*block*-poly(methoxydiethylene glycol acrylate) Diblock Copolymer with Thermoresponsive Behavior

**DOI:** 10.1021/acs.jpcb.5c06288

**Published:** 2025-11-13

**Authors:** S. Kripotou, A. Manochis, A. Miasnikova, A. Laschewsky, C. M. Papadakis, A. Kyritsis

**Affiliations:** † Department of Physics, 68994 National Technical University of Athens, Zografou Campus, 15780 Athens, Greece; ‡ Electronic Devices and Materials Lab, Department of Electrical and Electronics Eng., 563186 University of West Attica, 12244 Athens, Greece; § 26583 Universität Potsdam, Institut für Chemie, Karl-Liebknecht-Straße 24-25, 14476 Potsdam-Golm, Germany; ∥ Fraunhofer Institut für Angewandte Polymerforschung, Geiselbergstr. 69, 14476 Potsdam-Golm, Germany; ⊥ 84665 Technical University of Munich, TUM School of Natural Sciences, Physics Department, Soft Matter Physics Group, James-Franck-Straße 1, 85748 Garching, Germany

## Abstract

In this work, thermal transitions and dielectric behavior of mixtures of water with thermoresponsive polystyrene-*block*-poly­(methoxydiethylene glycol acrylate) (PS-*b*-PMDEGA) copolymers are studied in the low water concentration regime (0 ≤ *h*_w_ ≤ 0.53). By gradually increasing the water content, the plasticization of the polymer matrix can be followed up to water contents around 0.30. At this critical water content, water crystallizes during cooling, and surprisingly, the glass transition temperature shifts abruptly to higher values becoming equal to that of a dry copolymer. This finding indicates that the water phase separation during the formation of ice crystals is accompanied by a water detaching process that leads to a complete rearrangement of PMDEGA macromolecular chains, similar to what happens during the water detaching process in the thermoresponsive coil-to-globule transition of the copolymer in aqueous solutions. Differential scanning calorimetry (DSC) measurements reveal a transition that resembles the thermoresponsive transition in aqueous solutions that takes place only for water content *h*_w_ > 0.30. For lower water contents, broadband dielectric spectroscopy (BDS) measurements indicate that uncrystallized water, weakly interacting with the PMDEGA block, may adopt an open hydrogen bond (HB) network structure of a solid-like structure with a characteristic time scale slower than hexagonal ice. Moreover, the observed crossover of the dynamics of secondary (or fast) water relaxation at about −90 °C (in the form of fragile-to-strong transition) may reflect an inherent property of HB network of hydration (interfacial/confined) water molecules that undergo changes in the structure and/or dynamics that may trigger the micro-Brownian mobility of the surrounding polymeric segments.

## Graphical abstract

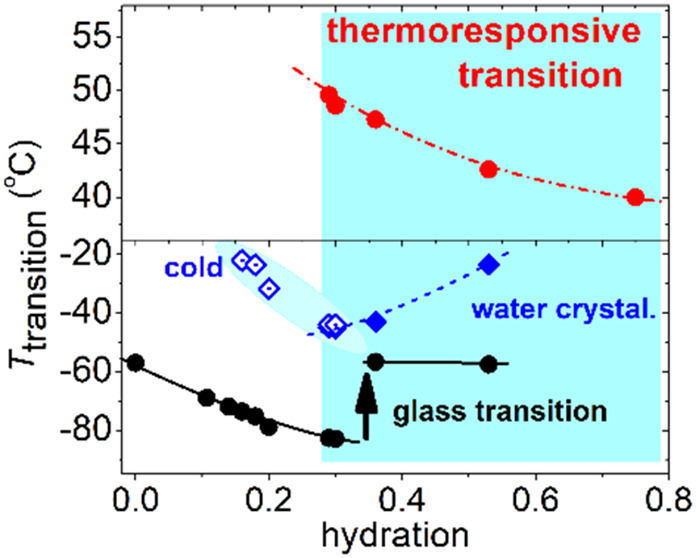

## 1 Introduction

Stimuli-responsive polymers represent a special class of soft materials that have attracted strong attention due to their numerous applications as drug delivery systems, templates for tissue engineering, sensors, and switches.^1–6^ In particular, the thermoresponsive polymers, i.e., the polymers that react strongly to small temperature perturbations, are in the focus of many research efforts due to various eventual applications and because they may be considered as prototypes for many biological macromolecules as well.^7–11^ The thermoresponsive polymers form a homogeneous mixture with a particular solvent (in most cases, with water) below or above a critical temperature. Upon crossing this critical temperature, by either heating (LCST) or cooling (UCST), phase separation into polymer-rich and polymer-poor phases occurs.

The key factor for the thermoresponsive behavior of a macromolecule is the amphiphilic character of its repeating unit. The monomeric unit or the macromolecular structure shall feature both hydrophilic and hydrophobic groups so that attractive and repulsive interactions coexist between the polymeric segments and water molecules (considering water as the solvent here). The balance between these forces at each temperature determines the state of the mixture: either one phase of a homogeneous mixture or a two-phase system. Cooperativity has also been identified as a key factor in the thermoresponsive behavior, as evidenced by comparative studies between PNIPAM polymer and NIPAM monomer.^12,13^ Different approaches have attempted to explain the origin of the thermoresponsive behavior.^10,14^ The most plausible scenario is based on destabilization of the water structures around the hydrophobic moieties of the macromolecule. Water molecules establish hydrogen bonds with the hydrophilic groups and eventually they form a thin layer (hydration layer) surrounding these groups as suggested by Fourier transform infrared (FTIR) results.^15^ On the other hand, water molecules are organized in an ordered ice-like structure around the hydrophobic part of the polymer^16^ under repulsive forces. These ordered structures have been claimed to be clathrate-like structures, and the involved water molecules are called “cage” water molecules. During the demixing transition, the hydrogen bonding (HB) network of these “cage” water molecules is destroyed, and as a result, the hydrophobic segments associate and the chain collapses. Thus, the hydrophobic effects were dominant. In parallel, the hydration layers become unstable, and the population of the water molecules that interact directly with the polymer is reduced after the reorganization of the whole hydrogen-bonded water network.

A class of poly­((oligoethylene glycol) [meth]­acrylate)­s that exhibits thermoresponsive behavior for short PEG side chains has been recently synthesized.^17–20^ Within the class of acrylates, poly­(methoxydiethylene glycol acrylate) (PMDEGA) has the shortest possible side chain to achieve water solubility, and it indeed exhibits pronounced thermoresponsive behavior. Thus, PMDEGA is sterically very close to the group of thermoresponsive poly­(*N*-alkylacrylamide)­s that are the most studied class of thermoresponsive polymers. Moreover, different from poly­(*N*-isopropylacrylamide) (PNIPAM), PMDEGA cannot form self-consistent inter- and intramolecular H bonds resulting in differences between the thermoresponsive behavior of the two types of polymers.^20^

Indeed, our previous studies on the thermoresponsive behavior of PS-*b*-PMDEGA polymer solutions reveal distinct features from similar PS-*b*-PNIPAM solutions: the temperature breadth of the transition in calorimetric studies is almost double in PS-*b*-PMDEGA solutions, whereas the organization of the expelled water during the macromolecular water detaching process and the subsequent evolution of micellar aggregates may be different. It was found that in the PS-*b*-PMDEGA solution, the water detaching process triggers the rearrangement of the whole PMDEGA blocks, leading to the formation of micellar aggregates, where a large part of the expelled water molecules is trapped and charge accumulation takes place. The size of these aggregates has been estimated to be on the order of 20–40 nm, depending on the temperature within the two-phase region. On the contrary, the demixing transition in the PS-*b*-PNIPAM solution is associated with a restriction of the local chain mobility of PNIPAM and the consequent reduction of the chain conformation modes (partial vitrification process).^21^ Papadakis’s group has intensively studied the swelling kinetics of initially dry thin films of PMDEGA homopolymers and triblock copolymers with PS end blocks as well as their dehydration kinetics employing in situ neutron reflectivity measurements. Additionally, the internal structure of these films was investigated with grazing-incidence small-angle X-ray scattering (GISAXS), while the bound water in the swollen film as well as the change of the bound water after the demixing process is probed by attenuated total reflectance Fourier transform infrared spectroscopy (ATR-FTIR).^22–24^ These studies reveal that although there are C=O and C–O moieties in the PMDEGA chains, water forms hydrogen bonds only with the C=O group. During the thermoresponsive transition, some hydrogen bonds break, and water is dissociated from the polymeric chains. However, not all hydrogen bonds are broken, meaning that there is still bound water trapped within the film. The finding that water molecules are mostly located near the hydrophobic backbone of the PMDEGA macromolecules may suggest that the particular organization of these confined water (“cage” water) molecules is the key point for the thermoresponsive behavior of PMDEGA. In this context, the investigation of the various forms of water organization within the PMDEGA matrix will help toward understanding the origin of the thermoresponsive transition of polymers.

Valuable insight into the organization of water molecules within the polymer matrix can be provided by investigating the hydrated polymers at subzero temperatures, where distinction can be made between uncrystallized and crystallized water fractions that reflect certain aspects of water–polymer interactions. Water molecules within a polymer matrix may remain liquid-like or nonfreezable at subzero temperatures when they are molecularly distributed around hydrophilic groups existing in the polymer structure (hydration sites). In this case, the polymer–water interactions dominate water–water interactions, and consequently, these water molecules are located around polar groups and may form a hydration shell under specific circumstances. These water molecules are considered the medium for the plasticization of the hydrated polymeric matrix. On the other hand, water molecules may also not be able to crystallize when they are confined within small volumes and their number is lower than the critical value for nucleation and ice formation.^25–27^ The properties of this so-called hydration water, either in the form of interfacial water or as confined water, depend strongly on the features of hydrogen bond (HB) networks where the water molecules participate and are intensively studied in the literature.^28–41^

A comparison of the properties of the hydration water in PMDEGA with those discussed in the literature is one of the objectives of this work. To that aim, we employ dielectric spectroscopy that has been proved to be a powerful tool for studying the dynamics of hydration water. Taking advantage of the fact that the dielectric response of water is very sensitive to the details of the HB network where water molecules are involved, many reports in the literature reveal the diversity of water HB networks that may be formed within a polymer matrix and try to express relationships with the underlying polymer–water interactions.^26,27,30,37,39,42–47^ For water contents above a critical threshold, the volume of the water in the polymer–water mixture exceeds a critical size, and phase separation occurs with the formation of the crystalline water phase. Thus, the water phase-separates below zero temperatures by forming the solid phase, and the question arises about the fraction of the water that remains uncrystallized in the mixture. In hydrophilic polymers and especially in biopolymers, it is known that the water molecules that are involved in the polymer–water interactions, called also bound water molecules, remain bound after the ice formation, i.e., only the excess water molecules participate in the crystalline water phase.^30^ Consequently, the glass transition of the polymer–water mixture is stabilized at low values due to plasticization from the bound water that remains molecularly distributed throughout the whole hydration range. On the other hand, the so-called regulation effect has also been observed in hydrated polymers.^48–50^ More specifically, when ice nuclei are formed above a critical water content, a fraction of water molecules that contributed to the plasticization of the polymer matrix phase join the ice crystals during the growth process. As a result, the polymer–water phase has less water than before the ice formation, and the glass transition rises to a value that corresponds to the new water content in the polymer–water phase (regulation effect). The amount of uncrystallized water is usually considered as a feature of each hydrophilic polymer and its structure/configurations. Polymer–water mixtures with partially crystallized water have been the subject of numerous research studies.^25,37,51–56^

In this work, we study the thermal transitions and dielectric behavior of polystyrene-*block*-poly­(methoxydiethylene glycol acrylate) copolymer–water mixtures within a broad concentration range (from dry to about 53% water fraction). As mentioned, for much higher water fractions (i.e., in dilute aqueous solution), the copolymers exhibit a thermoresponsive behavior.^21^ The measurements here are performed at subzero temperatures, and the study focuses on the water demixing process during ice formation. The discussion of the experimental results is based on the concepts of hydration water (interfacial or confined water) and water hydrogen bonding (HB) network in mixtures at various water contents, from low degrees of hydration, where the water molecules have strong plasticization action and do not phase-separate from the polymer, to high hydration levels, where water crystallizes during cooling and phase-separates. Furthermore, our results reveal that the water phase separation during the thermoresponsive transition in concentrated aqueous solutions and the ice formation at subzero temperatures share some similar characteristics.

## 2 Experimental Section

### 2.1 Materials

A diblock copolymer PS_11_-*b*-PMDEGA_513_ (Scheme 1) was synthesized as described previously.^57^ It has *M_n_* = 90,700 g/mol, as determined from end-group analysis by ^1^H NMR, and a dispersity index of 1.48. Hydrated samples with water fractions, *h*_w_, spanning from 0.1 to 0.53 were prepared as follows: A proper amount of copolymer and distilled water were mixed and left to equilibrate for 4 days at a subambient temperature (4 °C, in the fridge). The water fraction, *h*_w_, is defined as the mass of water relative to the total mass of the sample.

**Scheme 1:**
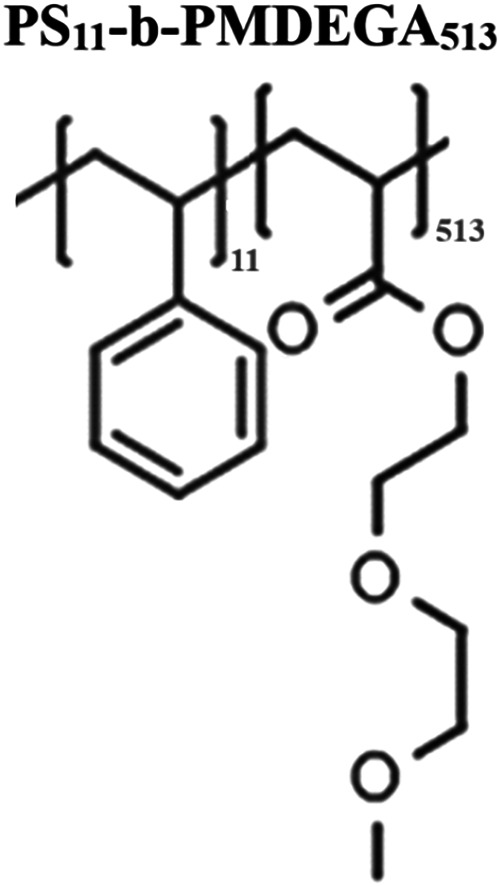
Chemical Structure of the Diblock Copolymer

### 2.2 Methods

#### 2.2.1 Differential Scanning Calorimetry (DSC)

For thermal characterization of the hydrated copolymer, a TA Q200 series DSC instrument calibrated with indium for the temperature and enthalpy was used. Experiments were performed with samples of similar mass (to avoid side effects of the sample size on the baseline signal) of 3–5 mg encapsulated in hermetically sealed aluminum pans under a nitrogen atmosphere. The samples were subjected to two thermal cycles of heating and cooling between −150 and 70 °C with a heating/cooling rate of 10 K/min.

The analysis of the DSC curves was performed using TA Universal Analysis software. The characteristics of the glass transition including the glass transition temperature, *T*_g_, and the constant volume heat capacity change, Δ*C*_p_, were calculated using the half height analysis. Melting and crystallization temperatures, *T*_m_ and *T*_c_, respectively, and their corresponding enthalpies were estimated from the integration of the peaks.

#### 2.2.2 Broadband Dielectric Spectroscopy (DRS)

For dielectric measurements, an α Analyzer in combination with a Quatro Cryosystem temperature controller and a BDS1200 sample holder (all provided by Novocontrol) were used. A round plate capacitor filled with the sample under investigation was prepared. The diameter of the capacitor electrodes was 20 mm, while the distance between them was kept constant at 50 μm using silica spacers of this diameter. The complex dielectric permittivity ε* = ε′ – *i*ε″ was recorded isothermally as a function of frequency, in a broad range from 10^–1^ to 10^6^ Hz at temperatures from −150 to 70 °C in steps of 5 °C.

Dielectric data were analyzed in the formalism of dielectric permittivity by using model functions in order to evaluate the characteristics of the recorded relaxation processes in terms of the time scale, dielectric strength, and shape. To that aim, we employed the asymmetric Havriliak–Negami (HN) equation for each relaxation process and an additional term for the dc conductivity, which results in the following expression for the imaginary part of dielectric permittivity, ε″(*f*)­

1 ε″(f)=∑i−Im[ε∞+Δεi(1+(if/f0i)αHNi)βHNi]+σ0ε∞2πf

where Δε is the dielectric strength, *f*_0_ is a characteristic frequency related to the frequency of maximum dielectric loss (ε″), ε_∞_ describes the value of the real part of dielectric permittivity, ε′, for *f* ≫ *f*_0_, and α_HN_ and β_HN_ are the shape parameters of the relaxation. The contribution of the dc conductivity to the ε″(*f*) response is represented by the last term in eq 1, where σ_0_ is the dc conductivity. A critical fitting of a sum of up to three HN terms to the experimental data at each temperature was performed. The number of terms needed was different for different hydration levels and temperatures. Due to high dc conductivity, the fitting procedure was also applied to the dielectric loss curves obtained from the derivative of the real part (eq 2), the so-called “conductivity free” approximation^58^

2 $$\varepsilon^{\prime \prime }\approx -\left(\pi /2\right)\frac{\partial \varepsilon^{\prime }\left(f\right)}{\partial \ln\left(f\right)}$$

## 3 Results and Discussion

### 3.1 DSC Results

This section presents and discusses the results obtained using differential scanning calorimetry (DSC). The thermograms of all of the investigated samples are first presented. Subsequently, a detailed discussion of the thermal transitions observed, including their characteristics, is provided with particular emphasis on the influence of water content on them.

Figure 1 shows the DSC cooling and heating curves of copolymers at various hydration levels. In Figure 1a,b, the thermograms obtained on copolymers with no crystallization events during cooling (*h*_w_ ≤ 0.20) are shown, whereas in Figure 1c,d, DSC curves obtained at higher hydration levels are presented, where water crystallization events are recorded during cooling. For all of the copolymers under investigation, a glass transition was observed as an endothermic step at subzero temperatures (Figure 1b,d), which is shifted to lower temperatures with increasing *h*_w_ (Figure 1b). According to our previous studies, this endothermic step is related to the glass transition of the PMDEGA block.^21,59^ During cooling, an exothermic peak was observed for water contents higher than 0.20 (*h*_w_ = 0.29 and higher, Figure 1c). Due to the absence of such a peak in the dry sample, this event is attributed to the crystallization of water within the polymeric matrix. Furthermore, while no crystallization of water occurs during cooling for water contents lower than 0.29, crystallization takes place during heating (cold crystallization effect) above the glass transition temperature for copolymers with water content *h*_w_ ≥ 0.16 (Figure 1b). In hydrated copolymers with water content 0.29 ≤ *h*_w_ ≤ 0.36, crystallization of water takes place during cooling at temperatures below −40 °C (−45 to −43 °C), thus exhibiting strong supercooling. Only for the copolymer with the highest water content (*h*_w_ = 0.53), water crystallization occurs during cooling in the temperature region around −20 °C, where the formation of hexagonal ice in highly hydrated polymers is usually recorded.^30,37,52^ Melting of ice formed during either cooling or heating is observed as an endothermic peak in the heating thermograms (Figure 1b,d). Furthermore, for copolymers with water content *h*_w_ ≥ 0.29, a peak that is exothermic upon cooling and endothermic upon heating, indicating a reversible thermal event that resembles the thermoresponsive transition of the copolymer, is observed in Figure 1c,d, respectively, at temperatures above 0 °C (indicated by the arrows).

**Figure 1:**
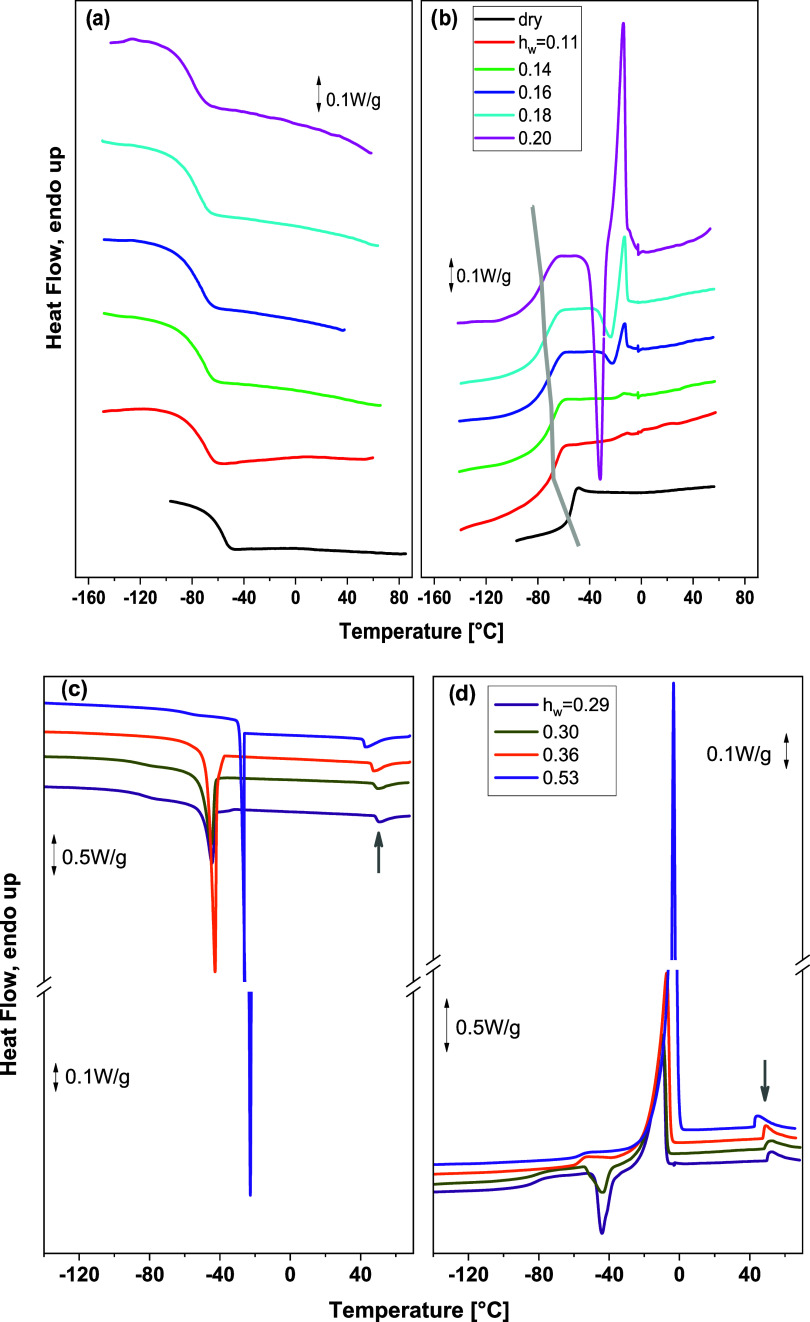
Heat flow as a function of temperature during cooling (a, c) and heating (b, d) with a rate of 10 °C/min for the samples with *h*_w_ ≤ 0.20 (a, b) and with 0.20 < *h*_w_ ≤ 0.53 (c, d). The curves have been shifted vertically for clarity. The arrows in panels (c, d) indicate reversible transitions due to the thermoresponsive behavior of the copolymer. The curved arrow in panel (b) is drawn to illustrate the shift of the glass transition step to lower temperatures with increasing water content.

In Figure 2, the characteristic temperatures of thermal transitions observed are shown as a function of the water content (phase diagram). As can be seen in Figure 2, *T*_g_ decreases from −57 to −83 °C with the water content *h*_w_ increasing up to 0.30, indicating a plasticization effect of water. As can be seen in Figure 2, these experimental data for water contents up to *h*_w_ = 0.30 are described well by the Fox equation^60^ (eq 3), using the value of −137 °C for the *T*_g_ of bulk water

3 $$\frac{1}{T_{g}}=\frac{w_{\left(\mathrm{poldry}\right)}}{T_{g,\left(\mathrm{poldry}\right)}}+\frac{w_{\left(\mathrm{water}\right)}}{T_{g,\left(\mathrm{water}\right)}}$$

A striking result is that for the two highest water contents (0.36 and 0.53), where crystallization of water takes place solely during cooling, *T*_g_ presents an abrupt deviation from the Fox curve shifting to higher values and becoming equal to that of the dry sample (Figure 2).^31^ Furthermore, in specific experiments where the cold crystallization was completed in a first heating scan and the specimens were cooled to −150 °C prior to ice melting, the *T*_g_ values that are obtained during the second subsequent heating scan have been found to approach or to be equal to the one of the dry copolymer. Details about this experiment can be found in Figure S1. These results indicate that the water phase separation during the formation of the ice crystals is accompanied by a water detaching process that leads to a complete rearrangement of PMDEGA macromolecular chains and the adoption of chain conformations similar to that in the dry state. The cold crystallization temperature decreases from −20 to −45 °C when the water content increases from 0.16 to 0.30 and follows the decrease of *T*_g_. Obviously, the enhanced molecular mobility of the copolymer at temperatures above *T*_g_ triggers the self-diffusion of adsorbed water, leading to the formation of water crystals within the samples. For water contents higher than 0.30, the crystallization temperature, *T*_c_, increases with increasing water content and reaches −20 °C for the highest water content studied (*h*_w_ = 0.53). The melting temperature, *T*_m_, is slightly lower than −10 °C for the samples where only cold crystallization is observed, while for those where crystallization occurs either during cooling and heating or solely during cooling, *T*_m_ increases with increasing water content reaching a value close to 0 °C for the sample with the highest water content (*h*_w_ = 0.53). This behavior implies that the ice crystallites formed exclusively during heating are smaller and/or contain an increased number of defects compared to those formed during cooling, and consequently, they melt at lower temperatures.

**Figure 2:**
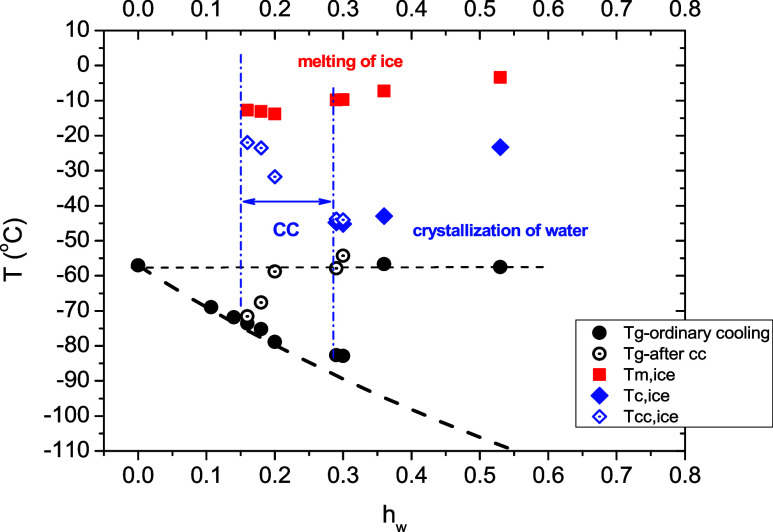
Glass transition temperature obtained during first heating, *T*_g_, and after completion of crystallization during heating, *T*_g–aftercc_ (see text for details), temperature of crystallization peak during heating (cold crystallization), *T*_cc,ice_, and during cooling, *T*_c,ice_, and temperature of ice melting peak, *T*_m,ice_, as a function of water content. The heavy black dashed curve represents the Fox equation considering the *T*_g_ values of water and dry copolymer equal to −137 and −57 °C, respectively.

In Figure 3, the recorded enthalpy of crystallization, Δ*H*, during cooling as well as during heating and the enthalpy of melting are presented as a function of the water content. For the samples where only cold crystallization occurs (for *h*_w_ up to 0.20), Δ*H*_melting_ is equal to Δ*H*_cryst_, while for the samples where crystallization takes place solely during cooling or in addition during heating, Δ*H*_melting_ is higher than the total Δ*H*_cryst_. The difference between these values decreases with increasing water content, and the two values are almost identical for the sample with the highest water content. This result may indicate that an ice thickening process takes place for the ice crystallites that are formed at the intermediate hydration levels (0.29 ≤ *h*_w_ ≤ 0.36). Moreover, the data in Figure 3 point to a critical water content for the water phase separation and the formation of the ice phase that is rather low, namely, around 10%. From the values presented in Figure 3, the fractions of crystallized and uncrystallized water within the samples were calculated and presented as a function of the water content in Figure 4. The crystallized water fraction, *X*_cw_, was determined from the recorded ice melting enthalpy, Δ*H*_m_, taking into account the reported value for bulk ice^61^ Δ*H*_mo_ = 334 J/g and applying the following equation

4 $$X_{\mathrm{cw}}\;\left(\%\right)=\frac{{\Delta H}_{m}}{h_{w}{\Delta H}_{\mathrm{mo}}}\times 100$$

The fraction of uncrystallized water is *X*_ucw_ (%) = 100-*X*_cw_ (%). Figure 4 shows that the crystallized water fraction increases with increasing water content, while there is always an almost constant amount of water (∼13% or *h*_w_ = 0.13) that remains uncrystallized within the samples. We emphasize, however, that this uncrystallized water does not contribute to the plasticization of the polymer, as the *T*_g_ in copolymers with partially crystallized water is equal to that of the dry sample as discussed above (Figure 2).

**Figure 3:**
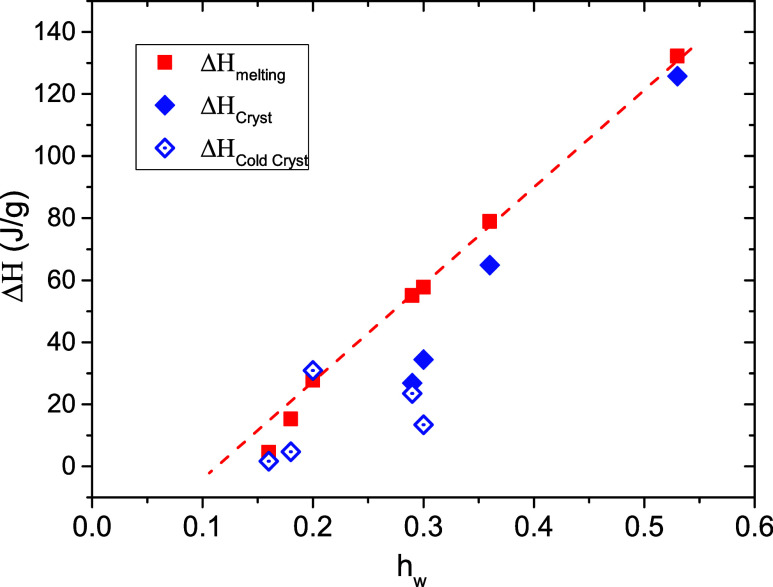
Enthalpy of melting, crystallization, and cold crystallization as a function of water content. Enthalpy values were obtained by integrating the area under the endothermic/exothermic peaks associated with the respective thermal transition.

**Figure 4:**
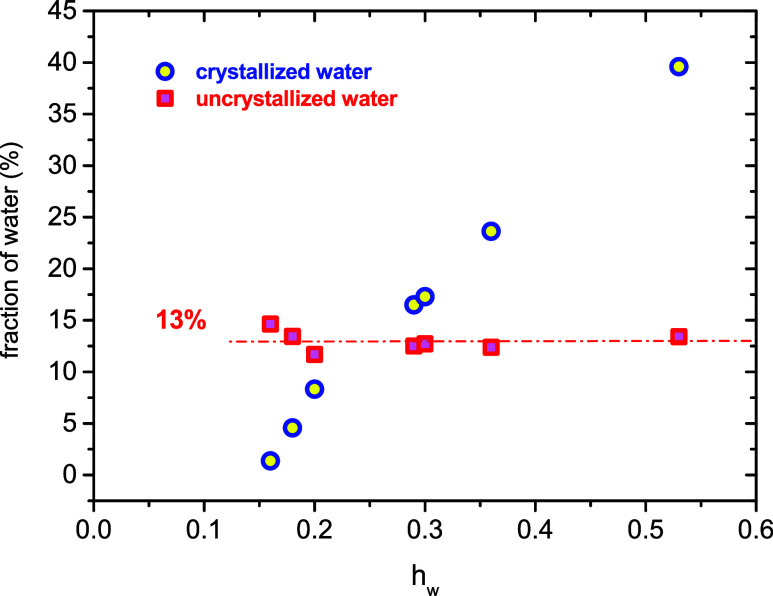
Fraction of crystallized and noncrystallized water as a function of water content.

As has been seen in Figure 1, apart from the glass transition and crystallization/melting of water, a transition that resembles the thermoresponsive transition in the aqueous solutions takes place above 0 °C in highly hydrated copolymers. This transition is observed as an asymmetric reversible peak for copolymers with water contents equal to or higher than 0.29 (Figure 5). The peak is water-content-dependent and shifts to lower temperatures with increasing water content, while a hysteresis between cooling and heating is observed. Transition takes place 2 °C lower during cooling than during heating regardless of the water content. This phase transition may be related to the thermoresponsive transition observed in aqueous solutions of the copolymer. Similar transitions were observed in hydrated gelatin samples that resemble the thermally induced sol–gel transition that occurs in gelatin macromolecules.^44,62^ Worth to note that in the copolymer under study, the thermoresponsive-like transition, i.e., the water detaching process during heating, takes place only for water content values where water can phase-separate and crystallize at subzero temperatures during cooling. We will come back to this point later.

**Figure 5:**
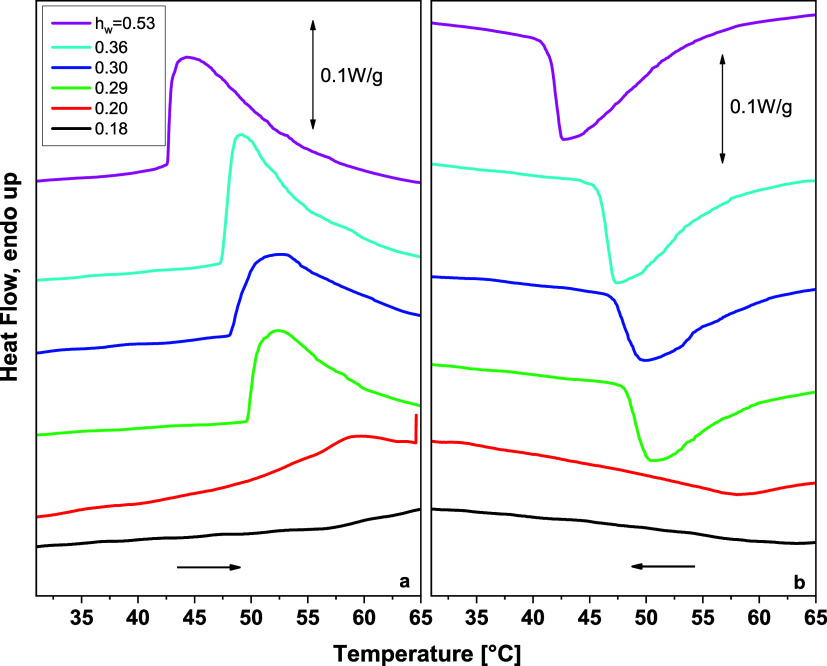
Heat flow as a function of temperature in the region of thermoresponsive-like transition during heating (a) and cooling (b) with a rate of 10 °C/min for all the samples indicated on the plot.

### 3.2 BDS Results

#### 3.2.1 Monitoring Thermal Transitions

Thermal transitions observed by DSC and discussed so far have also been recorded as changes in the real part of dielectric permittivity, ε′, in dielectric measurements. It is highlighted that in the case of first-order transitions, the change in the dielectric permittivity occurs at a specific temperature irrespective of the measuring frequency.^44^ In Figure 6, ε′ at 1 MHz as a function of temperature, during heating, for all of the hydrated copolymers studied by BDS has been plotted. Dielectric measurements have been recorded isothermally, and the data have been replotted in Figure 6 as a function of temperature at a constant frequency. For the dry sample, no transitions are observed apart from the dispersion due to the segmental α relaxation. For the hydrated systems, in order of increasing temperature, cold crystallization of water, melting of ice, and thermoresponsive-like transition, indicated by arrows in Figure 6, are followed. As can be seen, by increasing the temperature around −90 °C, ε′ starts to rise indicating an enhanced polarizability probably within liquid-like water structures. The increase of ε′ values stops suddenly, presumably due to water cold crystallization. Cold crystallization has been observed, for the hydrated copolymer studied by BDS, i.e., with *h*_w_ = 0.14, 0.16, 0.29, and 0.32. For the two lower *h*_w_ values, the event is recorded at −45 °C whereas for the two systems with higher *h*_w_, it is recorded at −60 °C (blue arrows in Figure 6). For the copolymer with *h*_w_ = 0.53, ε′ increases smoothly in the temperature range from −90 to −30 °C, indicating that no cold crystallization events occur, in agreement with DSC results. According to the DSC results presented previously, for water content *h*_w_ = 0.14 no water cold crystallization occurs, while for 0.18 and 0.29, the event takes place at −22 and −44 °C, respectively. This divergence between dielectric and calorimetric data, regarding the appearance or not of the cold crystallization and the temperature at which it takes place, can be understood taking into account the different thermal protocol applied at each technique and the kinetic character of the crystallization. In DSC measurements, the samples are heated at a constant rate of 10 °C/min, while BDS measurements are performed isothermally every 5 °C, which corresponds to a significantly lower heating rate (about 0.5 °C/min). Thus, melting of ice crystals, formed either during heating or during cooling is observed for all the hydrated systems studied by BDS. The hydrated sample with *h*_w_ = 0.53 does not show cold crystallization, so water crystals are formed solely during cooling (not followed by BDS). The temperature at which the drop in ε′ is observed (marked by red arrows) and is related to the melting of water crystals coincides with *T*_m_ obtained by DSC (Figure 2). In addition, it increases slightly with increasing water content. Thus, no significant difference between the values for *T*_m_ obtained by different techniques was found. The thermoresponsive-like transition is recorded as a frequency-independent change in ε′, the steep increase of its value indicated by black arrows,^21^ and is more pronounced in hydrated copolymer with *h*_w_ = 0.32 and 0.53 and less in the one with *h*_w_ = 0.29, while for 0.18 and 0.14, there is no indication in ε′(*T*) plots. The temperature at which thermoresponsive transition is observed by DSC (the peak temperature in Figure 1c,d) and BDS techniques differs only by 1–2 degrees.

**Figure 6:**
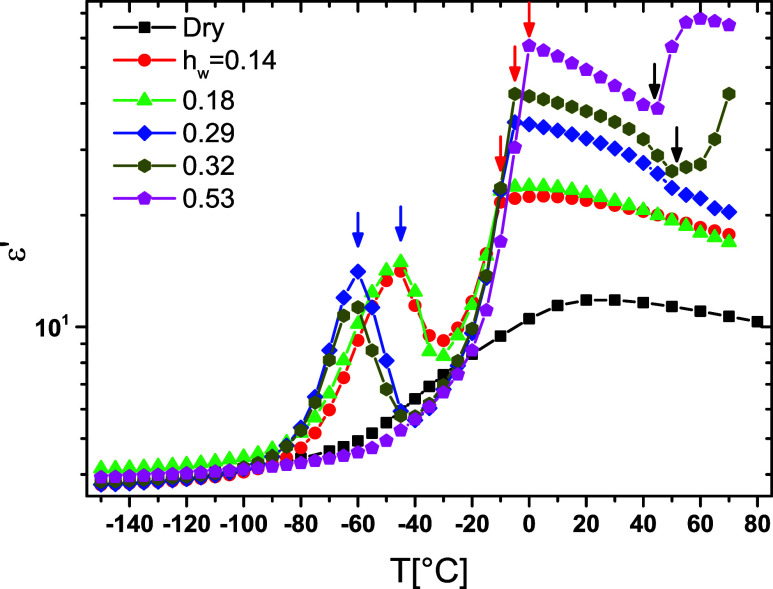
Real part of complex dielectric permittivity, ε′, as a function of temperature at 1 MHz for the samples indicated in the plot. The data have been recorded isothermally as a function of frequency and replotted as a function of temperature at a constant frequency.

#### 3.2.2 Molecular Mobility

In Figure 7, dielectric losses, ε″(f), for dry and hydrated copolymer are shown at −100 °C. At this temperature, according to DSC results, water is not crystallized for hydrated systems with 0.14 and 0.18 water content, whereas it may be partially crystallized for those with 0.29, 0.32, and surely for 0.53. For the dry sample, a double peak is observed, corresponding to secondary relaxations (not discussed further here). For the hydrated samples, peaks of higher intensity compared to those of the dry sample can be followed. For 0.14 and 0.18, a single broad peak is observed (called relaxation I), while for higher water contents a contribution at the low-frequency side of the main peak can be seen. For 0.32 this contribution is followed as a peak, called relaxation II, while for 0.29 it is observed only as a broadening at the low-frequency side of the main peak. The higher intensity of the peak in hydrated copolymers compared to the dry state suggests its association with a relaxation process where the water molecules may be involved. The position of the main peak depends slightly on the water content in the range 0.14 ≤ *h*_w_ ≤ 0.32. This behavior implies that relaxation process I may not be associated with the local relaxation process of the dry matrix whose dynamics is affected by association with absorbed water molecules. Numerous dielectric studies on hydrated polymers revealed that such a relaxation process (process I) is based on the local mobility of the matrix and is triggered and enhanced by the absorbed water molecules becoming faster and stronger with the increase of water content. The evolution of this low-temperature relaxation with increasing hydration level has revealed an interplay between a local dielectric relaxation mode of polar groups on the macromolecular chains interacting with water molecules and a relaxation process originated in the reorientation of water molecules themselves (the main secondary relaxation process of water or ν-process) above a critical water content.^63–67^ In the current case, the peak is observed at the same position for *h*_w_ = 0.14 and 0.18 as well as for *h*_w_ = 0.29 and 0.32. However, compared to the former hydrated systems, the peak in the latter systems is slightly shifted toward lower frequencies. The experimental finding that relaxation I is not *h*_w_-dependent points to a rather weak interaction among the polar groups of the copolymer and the added water molecules. The time scale of relaxation I is similar to the secondary process of water molecules themselves which has also been called the ν-process in the literature or fast water process^32,47,51,68,69^ and is related to the uncrystallized water within the copolymer. For the copolymer with the highest water fraction, 0.53, the dielectric dispersion may reflect mainly the dielectric response of the formed ice phase. Indeed, a double peak at higher frequencies, relative to all other hydrated copolymers, can be observed in the inset of Figure 7 for the sample with *h*_w_ = 0.53. The subscript “ice” is used to name the relaxations for the copolymer with *h*_w_ = 0.53 and to denote the existence of water crystallites at the low-temperature region.

**Figure 7:**
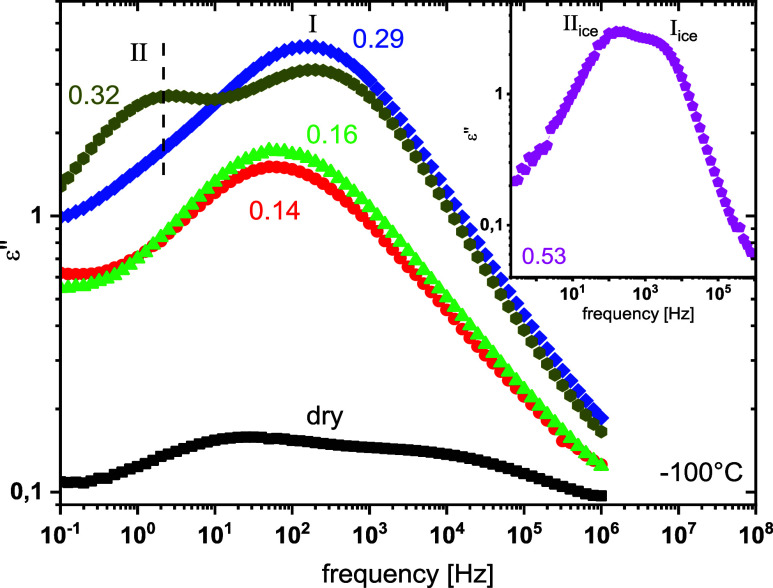
Imaginary part of complex dielectric permittivity, ε″, as a function of frequency at −100 °C for dry and hydrated samples, as indicated in the plot. In the inset, the spectrum obtained for the copolymer with *h*_w_ = 0.53 is shown.

The frequency dependence of the dielectric losses, ε″(*f*), at −35 °C for dry and hydrated copolymers is shown in Figure 8. For all of the systems presented in Figure 8, a single peak, called relaxation III, followed by a linear increase at low frequencies, can be observed. At this temperature range, water is partially crystallized within all the hydrated copolymers and relaxation III is assumed to be related to the formed ice phase. For the dry sample, the peak (at lower frequencies) is attributed to the main α relaxation associated with the glass transition of the sample (not discussed here). For the hydrated copolymers, the significantly larger intensity of the peak, compared to the one observed for the dry sample, indicates its relation to partially crystallized water. No significant dependence of peak position on water content up to 0.32 is observed, and only for the highest water content the peak, named II_ice_, is considerably shifted to higher frequencies, indicating a different morphology of the ice crystallites formed at the highest water content.

**Figure 8:**
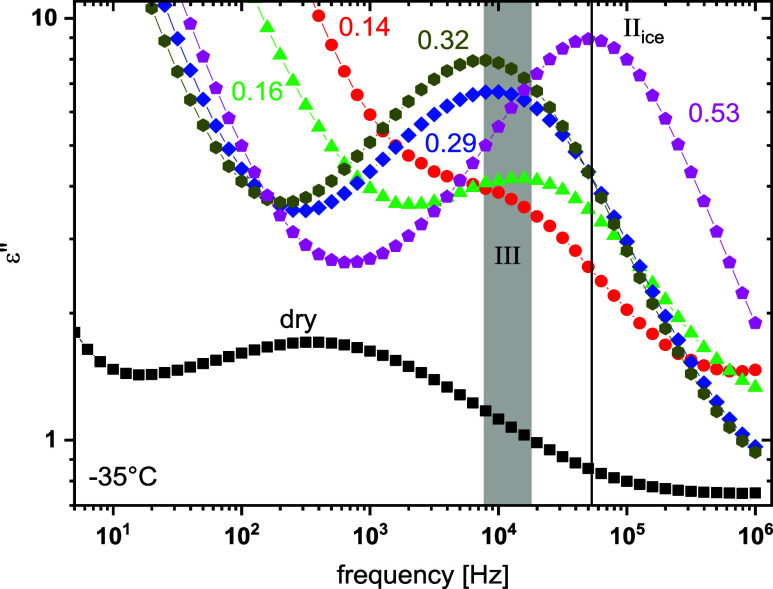
Imaginary part of complex dielectric permittivity, ε″, as a function of frequency at −35 °C for dry and hydrated systems. The vertical line and the shaded area indicate the peak frequencies for the hydrated system with *h*_w_ = 0.53 and *h*_w_ ≤ 0.32, respectively.

In order to extract information on the time scale and strength of the relaxations and to come into a discussion regarding the origin of the relaxations observed in hydrated systems, ε″(f) data were fitted using eq 1. For hydrated systems with *h*_w_ = 0.14 and 0.18, the peak of relaxation I is asymmetric up to about −75 °C and becomes symmetric at higher temperatures, while for the hydrated systems with 0.29 and 0.32, the peak remains symmetric in the whole temperature range. The shape parameter “a_HN_” is ∼0.5 for 0.14 and 0.18 and ∼0.6 for 0.29 and 0.32, indicating a broader distribution in the former case. Relaxations II and III are symmetric for all the systems, while the breadth of relaxation III is water-content-dependent. More specifically, the relaxation peak is broader for 0.14 and 0.18 (“a_HN_” ∼ 0.6–0.7) compared to the systems with higher water content (“a_HN_” ∼ 0.7–0.8). So, the corresponding distribution of relaxation time is water-content-dependent, being larger as the water content decreases. In the following, relaxations I–III will be discussed further in terms of their time scale and dielectric strength. First, the relaxations for the copolymer with water contents up to 0.32, i.e., without bulk-like ice, will be discussed, and then those for the copolymer with the highest water content (0.53).

##### 3.2.2.1 Copolymer with Water Contents up to *h*_w_ = 0.32

The Arrhenius plot of the relaxation time, τ, along with the dielectric relaxation strength, Δε, as a function of inverse temperature for relaxations I–III in the hydrated systems with water contents up to 0.32 is presented in Figure 9. Figure 9a shows the data for water contents of 0.14 and 0.18, where our DSC studies suggest that no ice is present down to −150 °C, whereas Figure 9b presents the Arrhenius plots for the data recorded on the copolymers with *h*_w_ = 0.29 and 0.32. It is important to note that for these water contents, strong supercooling effects were observed in the DSC measurements (Figure 1c).

**Figure 9:**
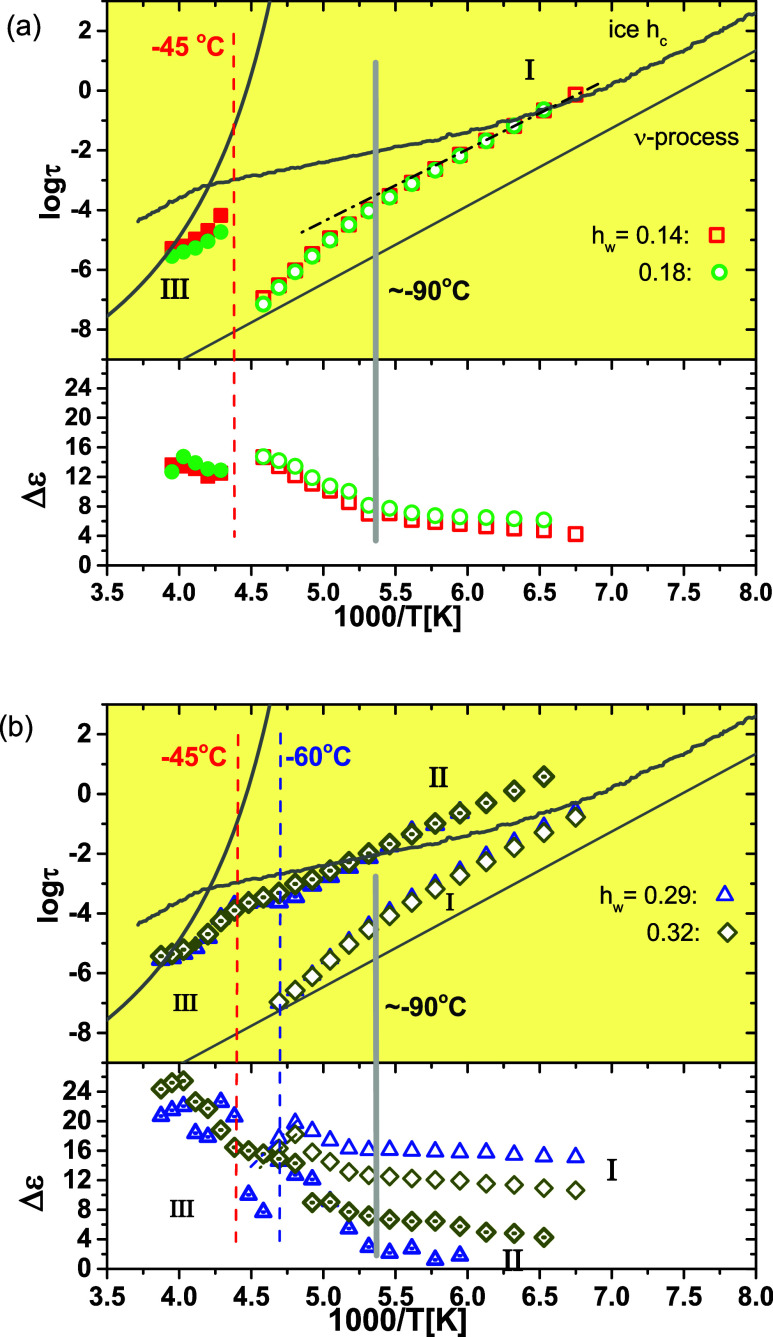
Arrhenius plot of the relaxation time (upper panel) and dielectric relaxation strength Δε (lower panel) for the relaxation processes followed in the copolymers with *h*_w_ ≤ 0.18 (a) and *h*_w_ = 0.29 and 0.32 (b) that exhibit cold crystallization events. The vertical dashed lines indicate the temperatures at which cold crystallization is observed by BDS. VTF curve describing the α relaxation of dry copolymer is included as a solid gray line.

As has already been discussed, **relaxation I** corresponds to the ν-process or secondary (fast) relaxation process of water. The temperature dependence of relaxation time of relaxation I demonstrates a dynamic crossover for all of the hydrated copolymers (Figure 9). The temperature at which the crossover occurs is around −90 °C (solid vertical line in Figure 9) almost independent of the water content. The crossover temperature is 10 and 5 °C lower than the *T*_g_ of the copolymer with water contents of 0.18 and 0.29, respectively. At temperatures below the crossover temperature, the temperature dependence of relaxation time follows Arrhenius behavior with a mean activation energy of 0.53–0.51 eV. Above the crossover temperature, an Arrhenius behavior is also followed for all the systems with a higher mean activation energy of 0.81–0.83 eV. Such a crossover around −90 ± 10 °C has been observed in the molecular dynamics of water in many hydrated systems, both under “soft” or “hard” confinement of water molecules, whereas the underlying molecular mechanism of this crossover is still under debate.^32,36,39,44,65,70–72^ One scenario describes this crossover transition like a fragile-to-strong transition by decreasing temperature, where the liquid-like behavior of confined water (VTF temperature dependence) changes to a solid-like behavior (Arrhenius temperature dependence) due to finite size effects imposed to confined water molecules. In the case of “soft” confinement, i.e., water mixtures with macromolecules, this dynamic crossover of water dynamics occurs at the same temperature range where structural rearrangements of the hosted macromolecules are frozen, leading to the liquid-to-glass transition of the hydrated system. Another approach is based on the concept that this transition from liquid-like behavior to the solid-like one is the manifestation of the liquid-to-glass transition for the interfacial or confined water.^39^ The crucial point in this approach is that the change of the (confined) water dynamics around −90 °C is an inherent characteristic of its hydrogen-bonded network and not dependent on the host material. Finally, a scenario that has been recently proposed relies on the framework of the Gibbs–Thomson theory and relates the crossover with the water crystallization that has been strongly suppressed due to small confining volumes.^26^

The temperature dependence of the activation energy (*E*_act_) of relaxation I is further examined by analyzing the experimental data. *E*_act_ as a function of temperature, derived from the derivative of the relaxation time in the Arrhenius plot, is shown in Figure S2. A continuous variation in *E*_act_ with temperature is observed, almost in the whole low-temperature range. A rather weak temperature dependence of *E*_act_ is observed up to −90 °C for *h*_w_ = 0.14–0.18 and up to −95 °C for *h*_w_ = 0.29–0.32, which is followed by a stronger temperature dependence in the interval −90 to −65 °C for *h*_w_ = 0.14–0.18 and from −95 to −70 °C for *h*_w_ = 0.29–0.32. By further increase of temperature, *E*_act_ decreases. Interestingly, this change in *E*_act_ follows the change of *C*_p_ in the region of the glass transition of the system, as recorded by DSC (Figure S2). A similar behavior in the derivative of the relaxation time has recently been observed in water solutions of ε-poly­(lysine).^72^ This result reveals the close relationship between the mobility of the uncrystallized water molecules (related to the ν-process) and the mobility of the polymeric matrix. In this context, it has been suggested that the onset of structural rearrangements of the matrix affects the mobility of hydration water due to dominant polymer–water interactions, e.g., appearance of the crossover of fast (ν) process and establishment of long-range proton mobility.^44^ However, in the current case, where the polymer–water interactions seem to be weak, it is reasonable to consider the possibility that a reverse causality relationship may hold, i.e., that changes in the water mobility, e.g., unfreezing of local modes of water mobility, induce the structural rearrangements of the polymeric matrix. This finding may consist of an additional manifestation of the coupling between water and polymer dynamics, originally introduced for the dynamics of hydrated proteins.^29,42,73^ Adopting such an interpretation for the crossover effect, i.e., that it is an inherent property of confined water,^34,39,40^ our data in Figure S2 suggest that the dynamics in the HB network of uncrystallized water molecules exhibit a rather continuous change at the low-temperature range with the existence of a critical temperature around −90 °C. Slightly faster dynamics were observed for 0.29 and 0.32 compared to 0.14 and 0.18 in the whole temperature range of relaxation I indicating a small dependence of this critical temperature on water content. Moreover, it is worth to notice that relaxation I in our systems here is slower, about more than one decade, than the secondary water process recorded in hydrated MCM41 ^74^ (hard confinement) and in hydrated hydrophilic macromolecules.^44,46,65^ Probably, secondary water relaxation I here is activated within an HB network that may be more open than in bulk liquid water, approaching that of hexagonal ice. We speculate that the finding that the uncrystallized water establishes only weak hydrogen bonding interactions with PMDEGA, if any, may play a role in the formation of this particular open structure of water HB network, which is not usually observed in hydrated hydrophilic polymers and biopolymers.

Regarding the dielectric strength, Δε, of relaxation I, a change in its temperature dependence is observed in Figure 9 for all of the hydrated copolymers in the crossover region (around −90 °C). Below the crossover, Δε increases slightly with increasing temperature, whereas above the crossover, a much stronger temperature dependence is observed. However, the temperature dependence of Δε is not monotonic for the copolymer with 0.29 and 0.32 water contents (Figure 9b): a decrease in Δε is observed starting at −60 °C. At higher temperatures, relaxation I shifts out of the measurement window, and no accurate information for Δε can be extracted. However, the dielectric loss spectra in this temperature range, i.e., above −60 °C, show a continuous decrease in losses at high frequencies with increasing temperature (Figure S3), implying that the strength of relaxation I gradually decreases with increasing temperature up to approximately −40 °C. As has already been discussed (Figure 6), in this temperature range, a frequency-independent change in ε′ is observed, which has been attributed to the cold crystallization of water within the copolymer, completing around −45 °C. A similar decrease in dielectric losses at high frequencies with increasing temperature up to −45 °C is also observed for the copolymers with 0.14 and 0.18 water content, though less pronounced (Figure S3), corresponding to the temperature range of water cold crystallization in these copolymers. These findings suggest that either the ice formation disrupts the water hydrogen bonding network associated with relaxation I (e.g., due to changes in the correlation of water molecules^56^) or that water molecules that initially contribute to relaxation I join the growing ice crystals and no longer contribute to relaxation I. Since DSC results show an increase in *T*_g_ toward the value of the dry copolymer after the completion of cold crystallization (Figure 2), along with a reduction in the amount of noncrystallized water, we may assume that the second scenario is the most likely, i.e., water crystallites are formed during heating, incorporating water molecules that previously contributed to relaxation I as uncrystallized molecules.

Additionally, Δε of relaxation I was found to be dependent on the water content. Equal values of Δε were found for water contents of 0.14 and 0.18, while for 0.29 and 0.32, 3-fold and 2-fold increases, respectively, were found below the crossover. The lower values of Δε found for 0.32 compared to 0.29 can be explained by assuming that the fraction of uncrystallized water is smaller in samples with *h*_w_ = 0.32 due to an increased fraction of the ice phase that may have been formed during cooling (as indicated by the previously discussed DSC results).

**Relaxation II** is a low-temperature relaxation that is activated in parallel with relaxation I only for water contents of 0.29 and 0.32 (Figure 9b), i.e., in copolymers where water crystallization under strong supercooling occurred during cooling. It exhibits an Arrhenius-like temperature dependence with an activation energy of 0.42 eV. At approximately −40 °C, a change in its dynamics is observed, with the temperature dependence becoming non-Arrhenius beyond this point. As shown in Figure 9, the crossover temperature coincides with the highest temperature at which the cold crystallization process still occurs in the hydrated copolymers. Therefore, we consider as a distinct process, termed relaxation III, the process recorded in the temperature region above −40 °C since the relaxation process is activated in the copolymers after serious changes in their morphology, i.e., where further water separation takes place and new ice crystals are formed. Regarding the dielectric strength of relaxation II, it can be observed in Figure 9b that for a water content of 0.29, Δε is considerably low up to −90 °C and increases significantly with increasing temperature up to −60 °C (the temperature where the cold crystallization process takes place in the hydrated copolymer). At higher temperatures, a scattering of the data is observed in the temperature interval −60 to −40 °C in parallel with significant changes in the corresponding Arrhenius plot (the water cold crystallization interval). For the water content of 0.32, a small increase of Δε with increasing temperature up to −85 °C is observed and a steeper increase for temperatures up to −60 °C, while in the temperature interval −60 to −40 °C, Δε remains almost constant. The striking result in Figure 9b is that the time scale of relaxation II for the copolymer with *h*_w_ = 0.32 exhibits almost unchanged temperature dependence from the low-temperature region up to the temperature range where cold crystallization is completed. Only for temperatures higher than the establishment of the ice crystals, i.e., around −40 °C, a crossover takes place. This result is an indication that this low-temperature relaxation II for water contents 0.29 and especially for 0.32 is related with a low-density structure (the time scale of relaxation II is slower than hexagonal ice as can be seen in Figure 9) of solid (crystalline) water phase. We remind the reader here that the DSC results (Figure 1) imply that water crystallization may have taken place during cooling with a rather strong supercooling (*T*_c_ = −43 °C) at these hydration levels. Relaxation II may be related with this particular water crystalline structure that may be considered as the precursor for the bulk-like water crystalline phase.

**Relaxation III** is detectable in all copolymers with water content in the range *h*_w_ = 0.14–0.32 for temperatures above −40 °C, i.e., the highest temperature where water cold crystallization occurs in the hydrated copolymers. Thus, relaxation III is activated in all copolymers where water is partially crystallized and the copolymers exhibit the same glass transition temperature, close to that of dry copolymers, i.e., *T*_g_ = −57 °C (Figure 2). It is interesting that the traces of its time scale in the Arrhenius plot of Figure 9 coincide for all of the copolymers, being distinct from that of dry copolymers, while the dielectric strength exhibits a water content dependence, being more intense in the hydrated copolymer with higher water contents. Because water is partially crystallized via cold crystallization in all systems up to 0.32 at temperatures above −40 °C, we propose that relaxation III, observed across all systems, is linked to the distinct crystalline structure of water produced during cold crystallization.

##### 3.2.2.2 Copolymer with *h*_w_ = 0.53 Water Content

For the copolymer with water content 0.53, identified by DSC results as the system with the highest crystalline fraction of water formed solely during cooling, two main relaxations, called **relaxations I_ice_ and II_ice_**, are observed as a bimodal peak in the low-temperature dielectric loss spectrum shown in Figure 7. Fitting procedure revealed the existence of two more relaxations, a fast one (relaxation I_ice,b_) with comparable time scale with relaxation I_ice_ and a slower one than relaxation II_ice_ (relaxation II_ice,b_) with the same time scale as relaxation II in the copolymer with water content 0.32, as can be seen in Figure 10, where the Arrhenius diagram of the relaxation time for all of the relaxations is presented. However, the strength of these two relaxations is considerably lower than that of relaxations I_ice_ and II_ice_ as can be seen in the lower panel of Figure 10. Relaxation I_ice_ follows the Arrhenius law with an activation energy of 0.36 eV up to −90 °C (vertical solid line in Figure 10), which is the temperature at which a crossover of dynamics was found in the systems with lower water contents, as has already been discussed. Above this temperature, the temperature dependence of relaxation time is very weak. The activation energy of relaxation I_ice_ up to −90 °C agrees well with those reported for interfacial water between the ice crystallites^36,44,76^

**Figure 10:**
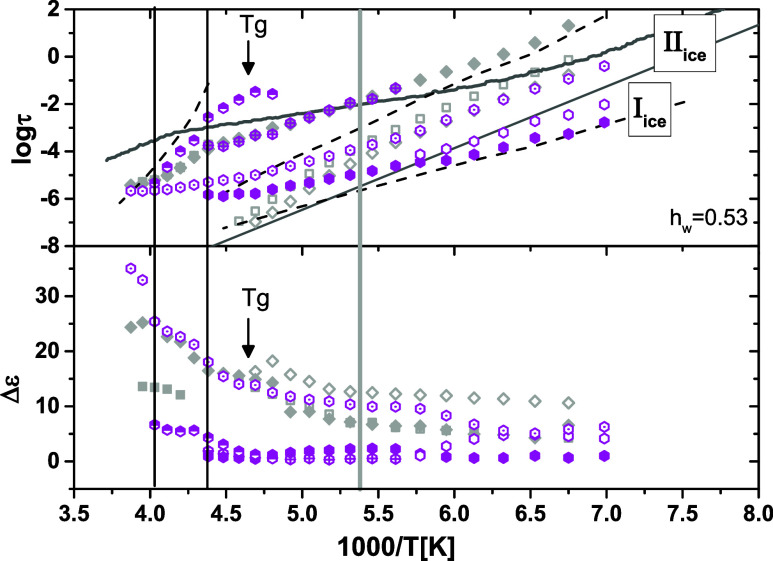
Arrhenius plot of the relaxation time (upper diagram) and dielectric relaxation strength, Δε (lower diagram), for the relaxations followed in the hydrated system with 0.53 water content. Data for *h*_w_ = 0.32 are included for comparison reasons.

Relaxation II_ice_ (presumably related to the main ice peak) has an activation energy equal to 0.4 eV, the same as relaxation II in copolymers with water content in the range of 0.29–0.32. However, the time scale of relaxation II_ice_ is almost 2 orders of magnitude faster as compared to that of relaxation II that is activated in the samples with lower water contents and strong water supercooling effects. Furthermore, above *T*_g_ (indicated by an arrow in Figure 10), the temperature dependence of its relaxation time becomes less intense (activation energy equal to 0.2 eV) whereas that of Δε becomes sharper. Our results reveal that the ice main peak (II_ice_) occurs at higher frequencies than hexagonal bulk ice, implying the existence of more defects than in bulk and/or a denser HB network of the solid phase of the water molecules.

#### 3.2.3 dc Conductivity in Hydrated Copolymers

In Figure 11, the temperature dependence of dc conductivity, σ_dc_, for dry as well as for all of the hydrated copolymers is presented. The values of σ_dc_ are obtained by the values of conductivity from the frequency-independent part of the conductivity spectra (dc plateau, Figure S4). For hydrated systems with *h*_w_ up to 0.32, a plateau in conductivity spectra is established only for temperatures higher than the temperature where crossover in dynamics of relaxation I was observed, i.e., −90 °C (black arrow in Figure 11). Below this temperature, no long-range charge mobility is possible since the polymer matrix is frozen and/or the water hydrogen bonding network seems to be noncontinuous. The temperature dependence of σ_dc_ up to the onset of cold crystallization (blue and red arrows) is VTF-like and resembles that of dry polymer shifted to lower temperatures. The onset of cold crystallization results in a decrease of σ_dc_, indicating either a decrease of charge concentration, a decrease in their mobility, or both. As has already been discussed, during cold crystallization, the water fraction taking part in the plasticization of the polymer matrix gradually decreases, and as a result, the polymer becomes stiffer and less mobile reaching the same *T*_g_ (*T*_g_ of dry copolymer) irrespective of the nominal water content. When cold crystallization has been completed (at −30 °C for 0.14 and 0.18 and at −40 °C for 0.29 and 0.32), the temperature dependence and the absolute values of σ_dc_ are independent of the water content up to the melting temperature of ice within the systems. In this temperature range, all traces in Figure 11 coincide, exhibiting rather an Arrhenius-like temperature dependence. The establishment of dc conductivity means that a continuous phase through which charge transport takes place has been formed. We assume that this dc conductivity process reflects the charge mobility within the existing uncrystallized fraction of water, which is constant, about 13%, independent of the initial water content. The rather high activation energy of about 1.0–1.1 eV for this long-range charge mobility has been observed also in other hydrated systems.^44,76^ It may be attributed to the particular HB network of this uncrystallized water and to the corresponding specific proton transfer mechanism.^77–79^ Recently, 2H NMR and BDS studies on water dynamics within amino-acid-functionalized silica nanopores^80,81^ reveal that when partial crystallization of water takes place the activation energy of the reorientation process increases from about 0.3 to ∼1.0 eV. It is speculated that the particular features of the liquid–solid water interfaces in partially crystallized nanoconfined water phases may be in the origin of the observed dynamics.^75^

**Figure 11:**
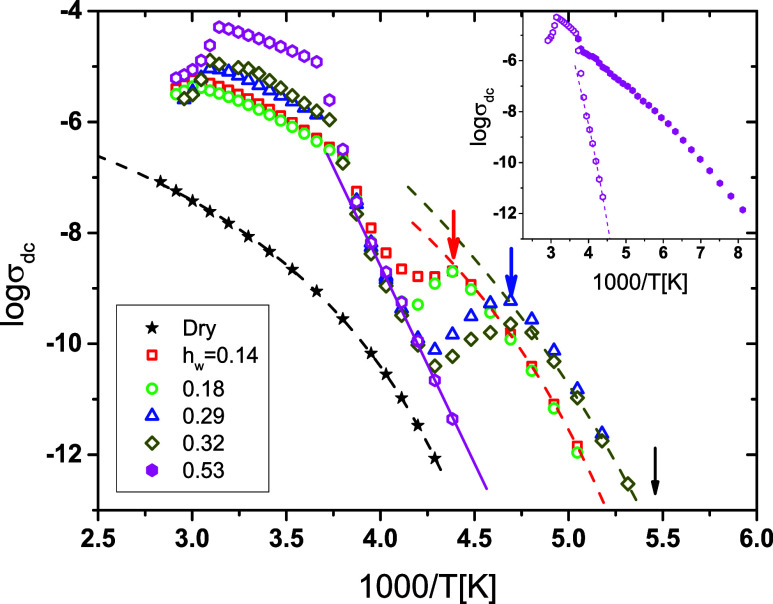
Arrhenius plot of dc conductivity, σ_dc_, for dry and hydrated systems. The black arrow denotes the temperature at which crossover of relaxation I dynamics was observed for systems with a water content up to 0.32. The blue and red arrows denote the temperatures at which cold crystallization of water takes place within the systems with 0.29 and 0.32 and 0.14 and 0.18 water contents, respectively. The dashed lines are fits of the data to VTF. The inset shows the corresponding Arrhenius plot for the hydrated copolymer with 0.53 water content (full symbols: values from the low-temperature conductivity plateau; open symbols: values from the high-temperature plateau).

The fact that the glass transition of the copolymers with partially crystallized water is the same, independent of the initial water content, suggests that the segmental mobility of the polymer matrix is similar in all hydrated copolymers affecting, thus, in the same way the protonic charge mobility. At temperatures above the melting of ice (starting for *T* > −25 °C, as estimated from BDS results presented above), σ_dc_ is water-content-dependent. The higher the water contents within the system, the higher the value of σ_dc_, which is a result of the increased number of mobile charges.

For the system with *h*_w_ = 0.53, a plateau in conductivity spectra can be followed at very low temperatures (Figure 11, raw ac conductivity data in Figure S4). dc conductivity at low temperatures follows an Arrhenius behavior with an activation energy comparable to that of relaxation I_ice_ within this system (0.33 eV). We speculate that the origin of this dc conductivity process is the amorphous water phase between ice crystallites that forms a percolating conductive cluster. These conductive paths are active up to the temperature range where ice crystals start to melt (at −25 °C). Interestingly, at temperatures close to −40 °C, a new long-range charge transport process is activated during heating, with much lower dc conductivity values. The trace of this process in the Arrhenius plot of Figure 11 coincides with the others of the copolymers with lower water contents. This result indicates that the uncrystallized water fraction provides the medium for the long-range charge mobility within the hydrated copolymers irrespective of the ice crystals morphology. It is also noted that in the copolymers with a water content of 0.53, the results clearly show that the onset of the low-dc value conductivity process is not related, at least directly, to the glass transition of the matrix (−57 °C).

Interestingly, for all the hydrated systems, a decrease in σ_dc_ is observed at the high-temperature range of the Arrhenius plot in Figure 11: at −60 °C for 0.14 and 0.18, at −50 °C for 0.29 and 0.32, and at −45 °C for 0.53. The decrease is sharp for high water contents (0.29, 0.32, 0.53) and smooth for 0.14 and 0.18. This effect is a result of the transition that takes place in the hydrated copolymers and is related to the thermoresponsive behavior of the copolymer.^21,59^

### 3.3 Particular Organization of Absorbed Water and Phase Separation

Our results indicate that the two-phase change transitions, i.e., the transition resembling the thermoresponsive transition at temperatures above 0 °C and the water phase separation/ice formation at subzero temperatures during cooling, occur for the same water content range (Figure 12). Furthermore, they share some common features. More specifically, they are both reversible and the water detaching process at subzero temperatures (for the ice formation) seems to drastically affect the conformations of the PMDEGA chains, as has been also suggested for the thermoresponsive/demixing phase transition in aqueous solutions of PMDEGA. Indeed, during water crystallization after the detaching process, a rearrangement of the whole PMDEGA blocks may take place and the segmental mobility is retarded, becoming similar to the polymers with no water molecules absorbed, i.e., no water molecules remain bound to the PMDEGA block, correspondingly affecting the segmental mobility and the glass transition. This behavior contradicts that recorded in many other hydrophilic polymers that are plasticized by water, and when water phase-separates to form ice, their *T*_g_ remains at low temperatures implying the continuous association with a fraction of water molecules that interact strongly with the polar groups of the polymer.^44,46,65^ Taking into account the finding that in the proximity of PMDEGA macromolecules, water molecules form preferential hydrogen bonds with the C=O group, it can be suggested that water molecules are accumulated close to the nonpolar main chain affecting the glass-to-liquid transition of the macromolecule by increasing the associated free volume rather than by interrupting polymer–polymer interactions. During the water phase separation, i.e., water crystallization, it seems that the water molecules join the ice nuclei being detached in a cooperative way, leaving the macromolecular chain empty of water. In particular, during the water cold crystallization process, the structural rearrangement of the polymer chains provides the trigger for the detaching of the water molecules and the formation of the solid ice phase. In this context, it is emphasized that although a fraction of uncrystallized water molecules (∼13%) exist in the copolymers after water crystallization, the glass transition of the copolymer with partially crystallized water is always similar to that in dry copolymers. Presumably, these water molecules are trapped in nanocavities within the polymer structure. Similarly, during the demixing phase transition of the thermoresponsive polymers (coil-to-globule transition), the globular macromolecules contain always an amount of water confined within the globular morphology.^23,24^ Worth noting that the water content of about 0.13, which remains uncrystallized, being, probably, confined in small voices, corresponds to one water molecule per MDEGA side chain, whereas water crystallizes during cooling at subzero temperatures and takes part in thermoresponsive-like transition around 40–50 °C for water contents higher than about 0.30 that corresponds, approximately, to 3 water molecules per MDEGA side chain.

**Figure 12:**
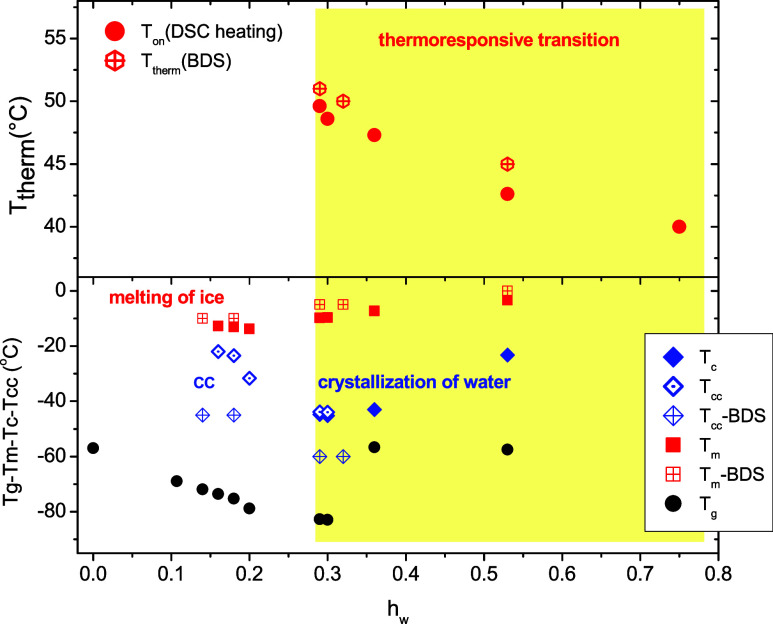
Thermoresponsive-like transition temperature obtained during heating by DSC and BDS (black arrows in Figure 6) as a function of water content (upper panel). Cold crystallization temperature, crystallization temperature, and melting temperature as a function of water content (lower panel). Data obtained by both DSC and BDS techniques are included in the diagram.

## 4 Conclusions

In this work, we study the thermal transitions and the dielectric behavior of mixtures of water with thermoresponsive polystyrene-*block*-poly­(methoxydiethylene glycol acrylate) copolymers in the low water concentration regime (from dry up to about 53% water fraction). By gradually increasing the water content, the plasticization of the polymer matrix can be followed up to water contents around 0.30. At this critical water content, water crystallizes during cooling, and surprisingly, the glass transition temperature shifts abruptly to higher values becoming equal to that of the dry copolymer. Thus, in partially crystallized samples, the Tg of PMDEGA block is at the value of the dry copolymer, although our DSC data imply that a fraction of about 13% of water remains uncrystallized.

In this rather low water content range of the water–copolymer mixture, DSC measurements reveal a transition that resembles the thermoresponsive transition in the aqueous solutions, that takes place only for water content values above a critical value (around 0.30), where water can phase-separate and crystallize at subzero temperatures during cooling. The critical water content value corresponds, approximately, to 3 water molecules per MDEGA side chain. Furthermore, our study suggests that the water phase separation during the thermoresponsive-like transition and the ice formation at subzero temperatures share some similar characteristics: (i) the water detaching process triggers the rearrangement of the whole PMDEGA block and (ii) a fraction of expelled water remains trapped within the micellar aggregates or the collapsed glassy polymeric chain conformations (about 13%).

Above the critical water content range (around 0.30) where water crystallizes during cooling, ice crystals with reduced sizes and/or high number of defects may be formed, which exhibit time scale for ice dipolar mobility faster than hexagonal ice. However, BDS measurements reveal that even at lower water contents, water molecules may adopt an open HB network structure of solid-like structure with the characteristic time scale (relaxation II) slower than the hexagonal ice. Our data suggest that this solid-like phase may be the precursor for the bulk-like ice that is formed at slightly higher water contents.

The recorded secondary (fast) water process, relaxation I, is attributed to the interfacial water molecules that surround, probably, the polymer backbone. The time scale of the process here is slower than that recorded in the hydrated hydrophilic polymers. Our BDS data support the claim that these water molecules easily join the ice nuclei during the growth of ice crystallites by being detached from the polymer chain after global rearrangement of the polymer chains during the glass-to-liquid transition. Interestingly, in the partially crystallized samples (after completion of the cold crystallization process, too), the dc (protonic) conductivity seems to have the same characteristic values, irrespective of the nominal water content exhibiting a temperature dependence with a rather high activation energy of about 1.0 eV. We speculate that the charge transport is activated via the uncrystallized water fraction.

Our measurements reveal the crossover of the dynamics of secondary (or fast) water relaxation (in the form of fragile-to-strong transition) at about −90 °C, a behavior that has been observed for the secondary relaxation of water in many hydrated systems. Taking into account that in the present systems our data suggest that no remarkable water–polymer interactions may exist and considering the finding that only at temperatures above this crossover temperature the polymer segments start to fluctuate, leading to structural rearrangements, according to the heat capacity measurements, we may consider the possibility that the crossover recorded at about −90 °C reflects an inherent property of the HB network of hydration (interfacial/confined) water. The probable changes in structure and/or dynamics of the hydration water may trigger the micro-Brownian mobility of the surrounded polymeric segments, as has been assumed within the framework of the water–polymer slaving process.

## Supplementary Material



## Notes

Glass transition of copolymer after the completion of cold crystallization, activation energy of relaxation I, dielectric loss spectra of relaxation I, and conductivity spectra (PDF)
