# Supplementary material for: Particular Water Organization in Homogeneous Water Mixtures of Amphiphilic Polystyrene-block-poly(methoxydiethylene glycol acrylate) Diblock Copolymer with Thermoresponsive Behavior
Source: J Phys Chem B. 2025 Nov 13;129(47):12306–20. doi: 10.1021/acs.jpcb.5c06288 (PMC12670404; doi:10.1021/acs.jpcb.5c06288)
Supplement: Supplementary file 1 [file jp5c06288_si_001.pdf]

Supporting Information

Particular Water Organization in Homogeneous Water Mixtures of Amphiphilic Polystyrene-*block*-Poly(methoxydiethylene glycol acrylate) Diblock Copolymer with Thermoresponsive Behaviour

S. Kripotou[1,2]*, A. Manochis*[1]*, A. Miasnikova*[3]*, A. Laschewsky*[3,4]*, C. M. Papadakis*[5]*, A. Kyritsis*[1]*

[1]*Department of Physics, National Technical University of Athens, Zografou Campus, 15780 Athens, Greece*

[2]*Electronic Devices and Materials Lab, Department of Electrical and Electronics Eng., University of West Attica, 12244, Athens, Greece*

[3]*Universität Potsdam, Institut für Chemie, Karl-Liebknecht-Straße 24-25, 14476 Potsdam-Golm, Germany*

[4]*Fraunhofer Institut für Angewandte Polymerforschung, Geiselbergstr. 69, 14476 Potsdam-Golm, Germany*

[5]*Technical University of Munich, TUM School of Natural Sciences, Physics Department, Soft Matter Physics Group, James-Franck-Straße 1, 85748 Garching, Germany*

*Corresponding Author: akyrits@central.ntua.gr

Glass transition of copolymer after the completion of cold crystallization

For the hydrated copolymer systems where, cold crystallization was observed during first heating (hw: 0.16 - 0.30), an additional experiment was performed by DSC technique. Cold crystallization was allowed to be completed (avoiding melting) and then the samples were cooled down to -150°C (10°C/min) and subsequently heated up to 70°C (10°C/min). The thermograms obtained during first heating and after the completion of cold crystallization are shown in the following Figure S1 as solid and dashed lines, respectively.

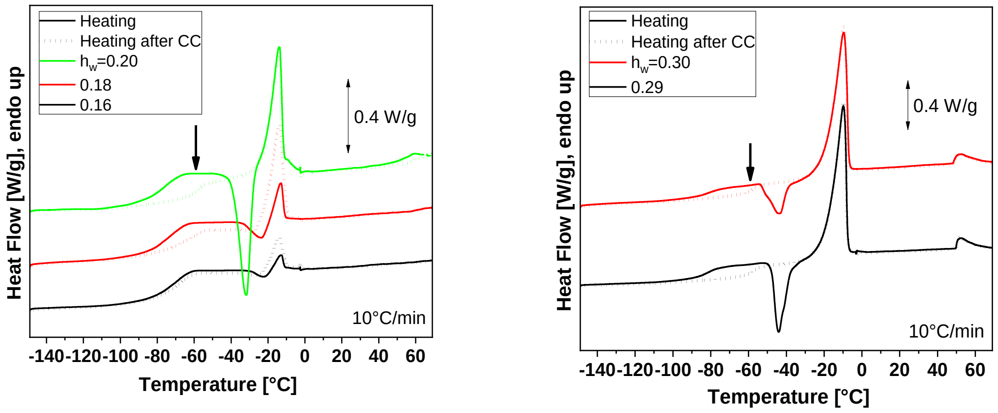

Figure S1. DSC thermograms for hydrated copolymer systems with h_w equal to 0.16, 0.18 and 0.20 (left panel) and with h_w equal to 0.29 and 0.30 (right panel) during first heating (solid lines) and after the completion of cold crystallization (dashed lines).

As can be seen in Figure S1, the crystallization of water results in a shift of the glass transition to higher temperatures towards that of the dry copolymer (arrow). Interestingly, the T_g value recorded for the system with hw=0.20 (Figure S1, left panel) is equal to the one of the dry copolymer, while for the systems with 0.29 and 0.30, it is even slightly higher (Figure S1, right panel) indicating a complete demixing of copolymer and water, which is caused by the crystallization. For the systems with h_w equal to 0.16 and 0.18, demixing is not complete as a result of incomplete crystallization, which is supported by the higher melting peak observed after cold crystallization compared to that observed at the first heating. For the latter systems,

melting starts immediately after cold crystallization completion, and it is difficult to achieve full completion of crystallization.

Activation energy of relaxation I

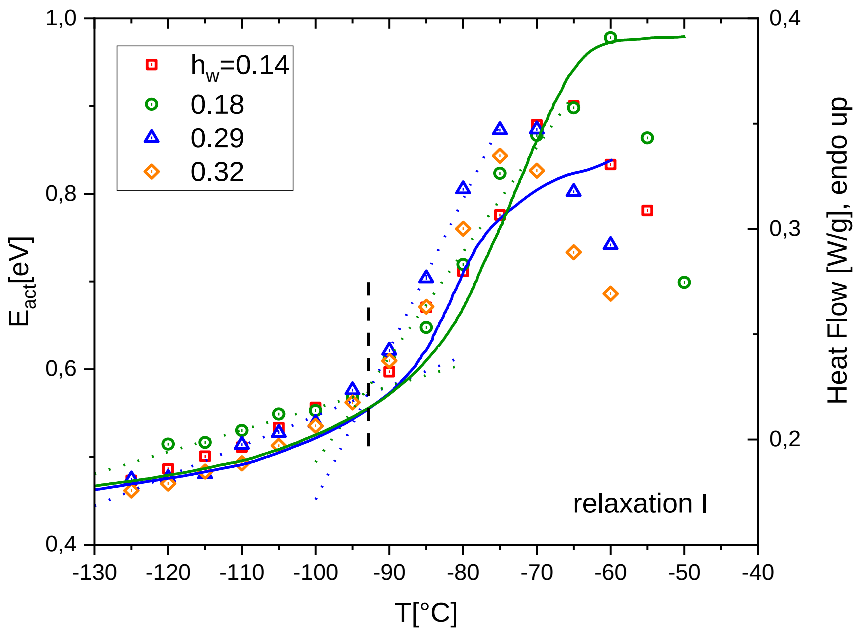

Figure S2. Activation energy, E_{act}, of relaxation I, determined from the temperature dependence of its relaxation time (symbols). Dotted lines represent linear fits to the data (low- and high-temperature domain). Solid lines represent DSC thermograms of hydrated systems with water content h_w of 0.18 and 0.29.

For all the hydrated systems E_{act} exhibits a temperature dependence, indicating deviation from ideal Arrhenius behaviour. This dependence is mild below -90°C and becomes more pronounced at higher temperatures. E_{act} reaches a maximum at -65°C and -73°C for systems with h_w=0.14 and 0.18, and h_w=0.29 and 0.32, respectively. The temperature dependence of E_{act} mirrors that of the heat flow, with the E_{act} maximum occurring upon completion of the glass transition step.

Dielectric loss spectra of relaxation I

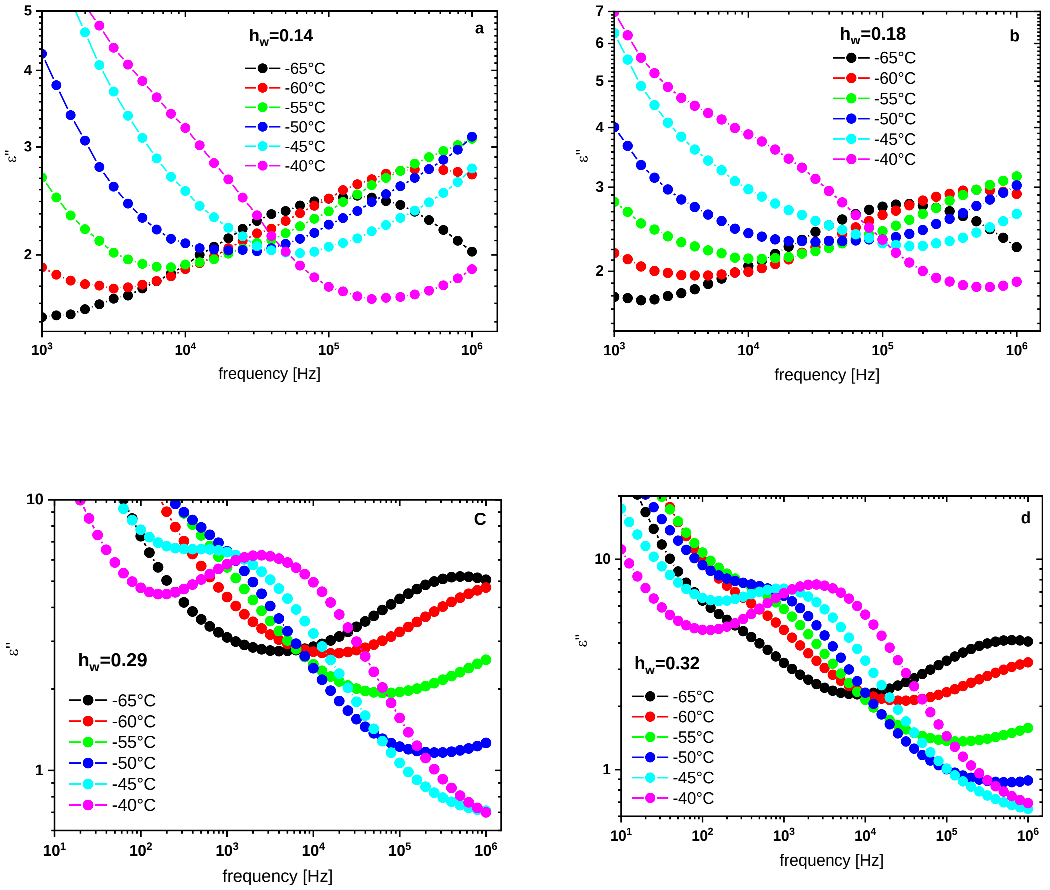

Figure S3. Dielectric losses, ε″, as a function of frequency at selected temperatures (indicated in the plot) for hydrated systems with water contents h_W= 0.14, 0.18, 0.29 and 0.32 shown in a, b, c and d, respectively.

The contribution of relaxation I on the high frequency side of the dielectric loss spectra decreases monotonically at temperatures ≥-45°C for hydrated copolymer with h_W=0.14 and 0.18 (Figure S3a, b) and at temperatures between -60°C and -40°C for those with h_W=0.29 and 0.32, respectively (Figure S3c, d).

Conductivity spectra

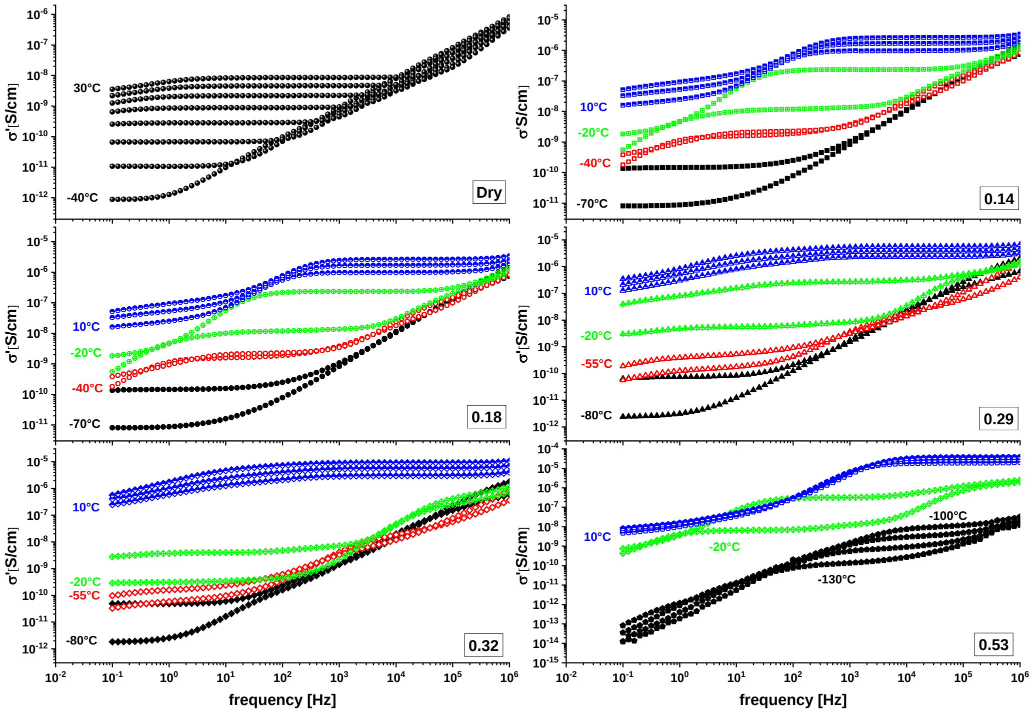

Figure S4. Conductivity spectra of dry and hydrated copolymer at selected temperatures. For the dry copolymer spectra are shown from -40°C to 30°C in 10°C increments. For hydrated copolymer with water contents h_W=0.14, 0.18, 0.29 and 0.32, different colors represent distinct temperature regimes: black curves correspond to temperatures below the cold crystallization temperature of water, T_{cc}, red indicate the cold crystallization region, green denote temperatures at which cold crystallization is complete, and blue correspond to temperatures above the melting temperature of water, T_m. For the copolymer with h_W=0.53, black curves represent temperatures below the glass transition temperature, T_g,, green indicate temperatures between T_g and T_m, and blue correspond to temperatures above T_m. In all cases, the temperature shown on the plot indicates the first curve of each color group, while subsequent curves differ by 10°C.
